# Perspectives on Tobacco Product Waste: A Survey of Framework Convention Alliance Members’ Knowledge, Attitudes, and Beliefs

**DOI:** 10.3390/ijerph120809683

**Published:** 2015-08-18

**Authors:** Sanas Javadian, Paula Stigler-Granados, Clifton Curtis, Francis Thompson, Laurent Huber, Thomas E. Novotny

**Affiliations:** 1 Graduate School of Public Health, San Diego State University, San Diego, CA 92182, USA; E-Mail: sanasj@gmail.com; 2 University of Texas School of Public Health, San Antonio, TX 77030, USA; E-Mail: paula.stigler@gmail.com; 3 The Cigarette Butt Pollution Project, Washington, DC 20004, USA; E-Mail: clifcurtisdc@gmail.com; 4 Framework Convention Alliance, Washington, DC 20004, USA; E-Mails: thompsonf@fctc.org (F.T.); huberl@fctc.org (L.H.); 5 Graduate School of Public Health, San Diego State University, San Diego, CA 92106, USA

**Keywords:** tobacco product waste, framework convention, cigarette butts

## Abstract

Cigarette butts (tobacco product waste (TPW)) are the single most collected item in environmental trash cleanups worldwide. This brief descriptive study used an online survey tool (Survey Monkey) to assess knowledge, attitudes, and beliefs among individuals representing the Framework Convention Alliance (FCA) about this issue. The FCA has about 350 members, including mainly non-governmental tobacco control advocacy groups that support implementation of the World Health Organization’s (WHO) Framework Convention on Tobacco Control (FCTC). Although the response rate (28%) was low, respondents represented countries from all six WHO regions. The majority (62%) have heard the term TPW, and nearly all (99%) considered TPW as an environmental harm. Most (77%) indicated that the tobacco industry should be responsible for TPW mitigation, and 72% felt that smokers should also be held responsible. This baseline information may inform future international discussions by the FCTC Conference of the Parties (COP) regarding environmental policies that may be addressed within FCTC obligations. Additional research is planned regarding the entire lifecycle of tobacco’s impact on the environment.

## 1. Introduction

Although tobacco use has enormous population health effects [1], its use also creates significant adverse environmental impacts due to tobacco agriculture and manufacturing [2]. In addition, discarded cigarette butts containing toxic chemicals (hereafter, tobacco product waste (TPW)) and are the leading item picked up during environmental cleanups around the globe [3]. This suggests that there may also be environmental damage from the six trillion cigarette butts that are smoked globally each year, with perhaps 2/3 deposited by smokers as litter (almost 800,000 MT of TPW) [4]. In one study, three-quarters of smokers reported a past history of tossing their cigarette butts on the ground or out of a car window [5]. Cigarette butts have been shown to leach out heavy metals into water, and it has been shown that one cigarette butt soaked in a liter of water for 96 hours is the Lethal Concentration 50 for test fish exposed to cigarette butt leachates [4]. Moreover, cigarette butts are made of cellulose acetate, which is a non-biodegradable plastic that collects chemicals produced by smoking [3]. This component of TPW may not degrade in the environment for many years, and even then may persist as small particles of toxic-infused plastic waste.

Numerous approaches to preventing and mitigating TPW have been suggested, such as developing deposit/return/take-back programs, labeling cigarettes as producing hazardous waste, applying litter fees to tobacco product sales, engaging in litigation to recover clean-up costs and alleviate public nuisance, levying fines for littering, banning the sale of filtered cigarettes, and educating consumers about TPW [6]. Anti-littering laws could reduce the number of cigarette butts deposited into the environment, but they are not strongly enforced and do not prevent the TPW waste stream. Further, these regulatory efforts are minimalist, downstream approaches when considering the enormity of global TPW. Research suggests a need for new approaches to TPW based on environmental principles, such as Polluter Pays, the Precautionary Principle, Extended Producer Responsibility (EPR), and Product Stewardship (PS) [7]. EPR is a strategy to decrease the environmental impact of a product by making the manufacturer of the product responsible for the entire life-cycle waste stream of product. PS overlaps with EPR, but calls for shared responsibility by all parties involved in the distribution and use of the product.

The Framework Convention on Tobacco Control (FCTC) is the first health treaty enacted under the authority of the World Health Organization (WHO); it entered into force in February 2005, and now has 180 Parties [8]. Articles 9, 18, and 19 of the Convention refer to tobacco-related environmental issues and to holding the tobacco industry responsible for tobacco harms. The Framework Convention Alliance (FCA) is an umbrella organization of civil society groups that support development, ratification, implementation, and monitoring of the FCTC (http://www.fctc.org/). Although the FCTC was negotiated and is executed by governments, the FCA continues to play an important role in the implementation process.

EPR and PS principles might be applied to TPW under the aforementioned FCTC Articles regarding the environment and tobacco industry responsibility. This descriptive study seeks to determine the level of knowledge, attitudes, and beliefs about TPW among FCA members in order to provide baseline data that might support future FCA policy work. Additional research is now being considered by the World Health Organization regarding the life-cycle environmental impacts of tobacco use, tobacco agriculture, tobacco manufacturing, and TPW [9]. The study was conducted by the Cigarette Butt Pollution Project (CBPP), a non-profit organization registered in California and a member of the FCA.

## 2. Methods

The study population was a convenience sample of FCA members obtained through the online survey tool, Survey Monkey [10]. The email listserv of FCA members was provided to CBPP by the FCA Secretariat in Washington, DC. Data collection was completed 6–27 February 2014. The survey had three sections: (1) knowledge and awareness of TPW; (2) general attitudes towards TPW and related environmental principles; and (3) demographic information about participants and their role in their organization/country. Questions were developed based on published TPW studies, such as by Rath *et al.* [5] and were pre-tested by CBPP volunteers. The online survey was administered according to FCA communication protocols. The study was approved by the Institutional Review Board (IRB) of San Diego State University. No incentives were offered for participation, and an informed consent statement was provided upon beginning the survey, indicating the voluntary and confidential nature of the study. Information collected was confidential but not anonymous, as we were interested in the types of organizations and membership status of participants. No explanations were provided about environmental principles queried (e.g., EPR and PS) in order to ascertain basic knowledge about these principles among the FCA representatives. The respondents to this survey provided individual-level responses rather than institutional positions.

After an initial email containing the survey link was sent on February 6, 2014, reminder emails were sent to the FCA members on February 13, 20, and 27. In the last two rounds, the survey was also provided in Spanish and French.

Simple frequencies and cross-tabulations are reported without statistical comparisons, as the response rate and sample size were not adequate for statistical analyses. Responses were stratified by WHO region.

## 3. Results

The response rate was 28% (n = 97). Most participants were male (60%), and the largest proportion (45%) were >50 years old. Respondents represented countries from all six WHO regions: Americas (26%), Europe (23%), Africa (22%), South Asia (12%), Middle East/North Africa (11%), and Western Pacific (6%). Among these, more than one-third (37%) has worked on tobacco control for more than 15 years, and the majority (64%) reported no previous affiliation with environmental advocacy groups.

Overall, a plurality (44%) identified cigarette butts as the most collected item in beach/roadway cleanups each year, with plastic bags/bottles/cups in second place (41%) (Table 1). Participants from Europe and the Western Pacific had the highest proportions citing TPW as the most collected item (68% and 63%, respectively), whereas those from Africa, Southeast Asia, and Middle East/North Africa identified ‘plastic’ as the most collected item (53%, 75%, and 55%, respectively). The majority of respondents (62%) have heard of the term ‘Tobacco Product Waste’ prior to taking the survey and almost all respondents (99%) considered TPW to be harmful to the environment. When asked who should be responsible for TPW clean up, most respondents (77%) indicated tobacco industry responsibility; they also indicated that smokers should be held responsible (72%). With respect to filters, the majority (86%) do not think filters make cigarettes less harmful and believe that cigarette filters are non-biodegradable (73%). Almost all (97%) of respondents believed that damage to the environment should be the greatest concern with regard to TPW. However, more than half of participants (54%) were not familiar with the environmental principles of EPR or PS, and 11% had never heard of these terms. Almost half of all respondents (48%) believed that EPR and PS should both be applied to TPW.

In additional cross-tabulations (data not shown in Table 1), among those who were familiar with EPR, only 27% felt that cities and communities should be responsible for TPW cleanup, whereas 82% of these indicated that the tobacco industry should be responsible. Among those familiar with EPR and PS principles, 89% were interested in applying articles 9, 18, and 19 of the FCTC to TPW. Among respondents who held smokers responsible for TPW, the majority (63%) did not feel well informed about TPW. However, most of them (71%) believed that their organization would be willing to address TPW as a tobacco control issue and that the FCTC is relevant to TPW. Among those who held cities/communities mainly responsible for TPW clean up, the majority (61%) did not feel well informed about TPW. Nevertheless, 61% believed that the issue of TPW is important in tobacco control.

**Table 1:** Knowledge, Attitudes, and Beliefs about TPW, Framework Convention Alliance Survey, by WHO Region, 2014.

Questions	Africa (*N* = 19)	Americas (*N* = 25)	Southeast Asia (*N* = 12)	Europe (*N* = 22)	Eastern Mediterranean (*N* = 11)	Western Pacific (*N* = 8)	Total (*N* = 97 respondents overall) **
The single most collected item in beach/roadway cleanups each year:							*N* = 97
Plastic (bags, bottles, cups)	52.6% (10)	36.0% (9)	75.0% (9)	18.2% (4)	54.5% (6)	25.0% (2)	41.2% (40)
Fishing line/nets	0.0% (0)	0.0% (0)	0.0% (0)	0.0% (0)	0.0% (0)	0.0% (0)	0.0% (0)
Cigarette butts	26.3% (5)	48.0% (12)	16.7% (2)	68.2% (15)	36.4% (4)	62.5% (5)	44.4% (43)
Aluminum cans	0.0%(0)	0.0% (0)	0.0% (0)	0.0% (0)	0.0% (0)	12.5% (1)	1.0% (1)
I don’t know	21.1% (4)	16.0% (4)	8.3% (1)	13.6% (3)	9.1% (1)	0.0% (0)	13.4% (13)
Have you heard of the term Tobacco Product Waste (TPW)?							*N* = 97
Yes	63.2% (12)	64.0% (16)	83.3% (10)	63.6% (14)	54.5% (6)	25.0% (2)	61.9% (60)
No	36.8% (7)	36.0% (9)	16.7% (2)	36.4% (8)	45.5% (5)	75.0% (6)	38.1% (37)
I don’t know	0.0% (0)	0.0% (0)	0.0% (0)	0.0% (0)	0.0% (0)	0.0% (0)	0.0% (0)
Do you feel well-informed about TPW?							*N* = 97
Yes	26.3% (5)	28.0% (7)	50.0% (6)	22.7% (5)	18.2% (2)	37.5% (3)	28.9% (28)
No	73.7% (14)	64.0% (16)	50.0% (6)	72.8% (16)	72.7% (8)	50.0% (4)	66.0% (64)
I don’t know	0.0% (0)	8.0% (2)	0.0% (0)	4.5% (1)	9.1% (1)	12.5% (1)	5.1% (5)
Do you consider TPW to be harmful to the environment?							*N* = 97
Yes	94.7% (18)	100.0% (25)	100.0% (11)	100.0% (22)	100.0% (12)	100.0% (8)	98.9% (96)
No	5.3% (1)	0.0% (0)	0.0% (0)	0.0% (0)	0.0% (0)	0.0% (0)	1.1% (1)
I don’t know	0.0% (0)	0.0% (0)	0.0% (0)	0.0% (0)	0.0% (0)	0.0% (0)	0.0% (0)
Do you believe that cigarette filters are biodegradable?							*N* = 96
Yes	21.1% (4)	0.0% (0)	25.0% (3)	4.8% (1)	18.2% (2)	0.0% (0)	10.4% (10)
No	52.6% (10)	76.0% (19)	66.7% (8)	90.4% (19)	63.6% (7)	87.5% (7)	72.9% (70)
I don’t know	26.3% (5)	24.0% (6)	8.3% (1)	4.8% (1)	18.2% (2)	12.5% (1)	16.7% (16)
Do you think that filters make cigarettes less harmful to smoke?							*N* = 97
Yes	10.5% (2)	4.0% (1)	16.7% (2)	9.1% (2)	18.2% (2)	0.0% (0)	9.3% (9)
No	78.9% (15)	96.0% (24)	83.3% (10)	86.4% (19)	63.6% (7)	100.0% (8)	85.6% (83)
I don’t know	10.5% (2)	0.0% (0)	0.0% (0)	4.5% (1)	18.2% (2)	0.0% (0)	5.1% (5)
In your opinion, how important is the issue of TPW in international tobacco control?							*N* = 90
Very important	88.8% (16)	21.7% (5)	63.6% (7)	38.1% (8)	70.0% (7)	71.4% (5)	53.3% (48)
Somewhat important	5.6% (1)	60.8% (14)	27.3% (3)	52.4% (11)	30.0% (3)	28.6% (2)	37.8% (34)
Not very important	5.6% (1)	17.3% (4)	0.0% (0)	9.5% (2)	0.0% (0)	0.0% (0)	7.8% (7)
I don’t know	0.0% (0)	0.0% (0)	9.1% (1)	0.0% (0)	0.0% (0)	0.0% (0)	1.1% (1)
Do you think your organization would be willing to address TPW as a tobacco control issue?							*N* = 89
Yes	88.8% (16)	54.5% (12)	91% (10)	47.6% (10)	80.0% (8)	71.4% (5)	68.5% (61)
No	0.0% (0)	0.0% (0)	0% (0)	0% (0)	10.0% (1)	14.3% (1)	2.2% (2)
I don’t know	11.2% (2)	45.5% (10)	9% (1)	52.4% (11)	10.0% (1)	14.3% (1)	29.3% (26)
Are you aware of TPW clean-up efforts in your country?							*N* = 90
Yes	22.2% (4)	39.2% (9)	18% (2)	42.9% (9)	20.0% (2)	14.3% (1)	30.0% (27)
No	72.2% (13)	56.5% (13)	82% (9)	52.4% (11)	70.0% (7)	85.7% (6)	65.6% (59)
I don’t know	5.6% (1)	4.3% (1)	%0 (0)	4.7% (1)	10.0% (1)	0.0% (0)	4.4% (4)
Who do you think should be responsible for TPW clean up *?							*N* = 90
Smokers	57.9% (11)	72.0% (18)	81.8% (9)	59.1% (13)	66.7% (8)	75.0% (6)	72.2% (65)
Cities/Communities	68.4% (13)	60.0% (15)	72.7% (8)	54.5% (12)	41.7% (5)	37.5% (3)	62.2% (56)
The Tobacco Industry	68.4% (13)	76.0% (19)	72.7% (8)	77.3% (17)	58.3% (7)	62.5% (5)	76.7% (69)
I don’t know	0.0% (0)	0.0% (0)	0.0% (0)	0.0% (0)	0.0% (0)	0.0% (0)	0.0% (0)
Are you familiar with the environmental principles of EPR and/or PS?							*N* = 89
Yes, I am familiar with both	27.8% (5)	21.7% (5)	20.0% (2)	9.5% (2)	20.0% (2)	28.6% (2)	20.2% (18)
Only EPR	0.0% (0)	4.3% (1)	0.0% (0)	33.3% (7)	10.0% (1)	14.3% (1)	11.2% (10)
Only PS	0.0% (0)	4.3% (1)	0.0% (0)	4.8% (1)	10.0% (1)	0.0% (0)	3.4% (3)
Neither	61.1% (11)	56.5% (13)	80.0% (8)	47.6% (10)	30.0% (3)	42.9% (3)	53.9% (48)
I don’t know	11.1% (2)	13.0% (3)	0.0% (0)	4.8% (1)	30.0% (3)	14.3% (1)	11.2% (10)
Do you think that EPR and/or PS should be applied to TPW							*N* = 89
Yes, both EPR and PS	55.6% (10)	39.1% (9)	33.3% (3)	52.4% (11)	54.5% (6)	57.1% (4)	48.3% (43)
Only EPR	5.6% (1)	0.0% (0)	0.0% (0)	19.0% (4)	27.3% (3)	0.0% (0)	9% (8)
Only PS	0.0% (0)	0.0% (0)	0.0% (0)	4.8% (1)	0.0% (0)	0.0% (0)	1.1% (1)
Neither	0.0% (0)	0.0% (0)	0.0% (0)	0.0% (0)	0.0% (0)	0.0% (0)	0.0% (0)
I don’t know	38.9% (7)	60.9% (14)	66.7% (6)	23.8% (5)	18.2% (2)	42.9% (3)	41.6% (37)
Do you think the Framework Convention on Tobacco Control (FCTC) is relevant in addressing TPW?							*N* = 88
Yes	83.3% (15)	78.3% (18)	66.7% (6)	85.7% (18)	50.0% (5)	57.1% (4)	75.0% (66)
No	11.1% (2)	0.0% (0)	22.2% (2)	0.0% (0)	20.0% (2)	14.3% (1)	8.0% (7)
I don’t know	5.6% (1)	21.7% (5)	11.1% (1)	14.3% (3)	30.0% (3)	28.6% (2)	17.0% (15)
What do you think people should be concerned about most with regard to environmental aspects of TPW *?							*N* = 90
Damage to the environment	89.5% (17)	92.0% (23)	81.8% (9)	90.9% (20)	91.7% (11)	87.5% (7)	96.7% (87)
Harm to people	84.2% (16)	52.0% (13)	90.9% (10)	45.5% (10)	75.0% (9)	37.5% (3)	67.8% (61)
Costs	31.6% (6)	48.0% (12)	54.5% (6)	59.1% (13)	33.3% (4)	62.5% (5)	51.1% (46)
Other	10.5% (2)	16.0% (4)	9.1% (1)	0.0% (0)	25.0% (3)	12.5% (1)	12.2% (11)
I don’t know	0.0% (0)	0.0% (0)	0.0% (0)	0.0% (0)	0.0% (0)	0.0% (0)	0.0% (0)
As a member of the FCA, would you be interested in applying Articles 9, 18, and 19 of the FCTC to TPW?							*N* = 90
Yes	100.0% (18)	73.9% (17)	70.0% (7)	66.7% (14)	90.9% (10)	85.7% (6)	80.0% (72)
No	0.0% (0)	8.7% (2)	0.0% (0)	4.8% (1)	0.0% (0)	0.0% (0)	3.3% (3)
I don’t know	0.0% (0)	17.4% (4)	30.0% (3)	28.6% (6)	9.1% (1)	14.3% (1)	16.7% (15)

* More than one response possible; ** N’s listed separately for each question.

## 4. Discussion

This limited survey of FCA-affiliated individuals to an online survey finds that almost all considered TPW to be harmful to the environment. Most of them also felt that TPW was an important consideration in international tobacco control. Similar to the Rath *et al.* (2012) study using the *Research Now* panel (an online marketing data source to evaluate smokers’ and non-smokers’ knowledge and beliefs), the majority of our respondents also believed that cigarette butts are toxic and knew that cigarette butts are non-biodegradable [5] (butts do photo-degrade under ideal circumstances, however [11]).

Although the majority of most respondents believed that cigarette butts were the most collected item during environmental cleanups, those from Africa, Southeast Asia, and the Eastern Mediterranean regions identified ‘plastic’ to be the primary waste item collected. Although plastics are a large source of waste found in environmental cleanups worldwide, TPW has repeatedly been the most collected item [3]. Therefore, our findings suggest that more information about the magnitude of TPW is needed for FCA members in some regions. In fact, the majority of respondents in these same regions did not feel well-informed about TPW.

Although most respondents were not familiar with the environmental principles of EPR and PS, the majority of those who were familiar with them reported that TPW damage to the environment is of concern as well a potential for harm to people and costs to communities for TPW cleanup and damage. Such findings suggest that the FCA members may be interested in pursuing tobacco industry accountability for the environmental effects of TPW. Accordingly, FCA respondents cited the applicability of FCTC Articles 9, 18, and 19 to this issue, as they relate to regulation, liability, and protection of the environment and health of persons.

This study was certainly limited by the low response rate (28%), but the average email survey response rate globally is even less (only 24.8%) [12].We offered no incentives, which would have likely increased the response rate, and in addition, many FCA members may not have been comfortable with the three languages used in this exercise. Nonetheless, some important information was obtained by this initial limited query, and additional qualitative research among FCA members and other tobacco control advocates might yield more nuanced data. At a minimum, many respondents (and perhaps non-respondents) were introduced to some new terminology (EPR/PS) that may be helpful to them to influence future FCTC COP deliberations on environmental issues. They may have consulted online information about them when answering the survey questions, in fact.

## 5. Conclusions

Our findings that FCA-affiliated individual respondents do recognize the importance of TPW globally suggest that additional attention to the environmental impact of tobacco use may be appropriate for the future FCTC Conference of the Parties deliberations. In particular, given the concern expressed for industry responsibility for TPW, discussions related to Article 5.3 (dealing with tobacco industry influence) may be of interest.

Science-based strategies to mitigate, reduce, and prevent TPW have been proposed in various reports [3,4,6,7] and we herein offer some specific suggestions for implementation of relevant FCTC Articles for consideration by the COP (Box 1). These approaches may create opportunities for new partnerships with environmental groups and may lead to more effective upstream actions to address the global TPW problem. Additional research on the lifecycle of environmental issues regarding tobacco agriculture, manufacturing, use, and TPW disposal may be undertaken by the WHO Tobacco Free Initiative in order to support implementation of FCTC Articles 9, 18, and 19. This information may then be used as part of a broader educational effort for tobacco control advocates, COP representatives, and other policy makers on the importance of TPW and other environmental consequences of the tobacco epidemic.

Box 1 **Environmental approaches to Tobacco Product Waste (TPW) that may be addressed through implementation of the Framework Convention on Tobacco Control.**

(1) Extended producer responsibility and product stewardship: Shift responsibility to the tobacco industry and other parties in the life cycle of tobacco product sales and usage for cleanup, take-back, and final disposal of TPW, including administration and monitoring of such programs.
(2) Ban disposable filters: A sales ban would reduce a significant portion of TPW.
(3) Extend bans on public smoking to outdoor areas: These restrictions serve to change the social norm regarding smoking and may thus also reduce the burden of TPW in outdoor environments.
(4) Labeling: Regulatory agencies could require a label of sufficient size that simply states: “Cigarette filters are non-biodegradable toxic waste. Safe disposal should be required in accordance with law.” Additional information could also describe potential toxicity of TPW, methods for safe handling, and applicable fines for littering.
(5) Litigation: The tobacco industry could be held responsible for the environmental impacts associated with sales of their products, with lawsuits that may be based on negligence and nuisance-related wrongful conduct, for failure to take reasonable steps to prevent harm, or for protecting someone’s right to use and enjoy the environment.
(6) Litter fees: Local authorities may apply litter fees as part of a framework program to recover cleanup and abatement costs and to conduct public education.
(7) Deposit/Return: Cigarettes could be sold with a “butt deposit” to be refunded when the butts are returned to the vender.
(8) Waste fees: Advanced Recycling Fees (ARF) could be assessed at the point of purchase, and such fees can then help cover the costs of recycling and proper disposal of TPW.
(9) Fines: Fines may be levied by state and local communities for littering on roadways, beaches, parks, and other public spaces. Fines could also be levied against cigarette manufacturers based on the quantity of brand-specific cigarette waste found on cleanups.
(10) Changing social norms: Achieving the “endgame” for tobacco and TPW will involve extraordinary social normative changes in the smoking ritual itself. As smoking becomes less and less socially acceptable, TPW disposal into the environment should also become less acceptable.

Adapted from Curtis *et al*. 2014 [7].
